# Relationship of Living Conditions With Dietary Patterns Among Survivors of the Great East Japan Earthquake

**DOI:** 10.2188/jea.JE20130025

**Published:** 2013-09-05

**Authors:** Nobuo Nishi, Eiichi Yoshimura, Kazuko Ishikawa-Takata, Nobuyo Tsuboyama-Kasaoka, Tetsuya Kubota, Motohiko Miyachi, Shinkan Tokudome, Yukari Yokoyama, Kiyomi Sakata, Seiichiro Kobayashi, Akira Ogawa

**Affiliations:** 1 Center for International Collaboration and Partnership, National Institute of Health and Nutrition, Tokyo, Japan; 1 （独）国立健康・栄養研究所 国際産学連携センター; 2 Department of Nutritional Education, National Institute of Health and Nutrition, Tokyo, Japan; 2 （独）国立健康・栄養研究所 栄養教育研究部; 3 Department of Nutritional Epidemiology, National Institute of Health and Nutrition, Tokyo, Japan; 3 （独）国立健康・栄養研究所 栄養疫学研究部; 4 Department of Clinical Nutrition, National Institute of Health and Nutrition, Tokyo, Japan; 4 （独）国立健康・栄養研究所 臨床栄養研究部; 5 Department of Health Promotion and Exercise, National Institute of Health and Nutrition, Tokyo, Japan; 5 （独）国立健康・栄養研究所 健康増進研究部; 6 National Institute of Health and Nutrition, Tokyo, Japan; 6 （独）国立健康・栄養研究所; 7 Department of Hygiene and Preventive Medicine, School of Medicine, Iwate Medical University, Yahaba, Iwate, Japan; 7 岩手医科大学医学部衛生学公衆衛生学; 8 Department of Plastic and Reconstructive Surgery, School of Medicine, Iwate Medical University, Morioka, Japan; 8 岩手医科大学医学部形成外科学; 9 Iwate Medical University, Morioka, Japan; 9 岩手医科大学

**Keywords:** Great East Japan Earthquake, living conditions, dietary patterns

## Abstract

**Background:**

During the year after the Great East Japan Earthquake on March 11, 2011, the health conditions and lifestyles of survivors were extensively surveyed. We examined the relationship between living conditions and dietary pattern among survivors.

**Methods:**

A total of 10 466 survivors aged 18 years or older (25% of the population of that age in the area) participated in a survey of Iwate Prefecture. The average frequency of daily consumption of 8 food groups was determined by questionnaire. After excluding staple foods, which were consumed 3 times a day by 85% of participants, factor analysis was performed on 7 food groups among 9789 people (3795 men, 5994 women).

**Results:**

Factor analysis identified 2 dietary patterns—prudent and meat. The prudent dietary pattern is characterized by high intakes of fish and shellfish, soybean products, vegetables, fruit, and dairy products and was more evident among older participants and women. The meat dietary pattern is characterized by high intakes of meat and eggs and was more evident among younger participants and men. Age-adjusted multiple logistic regression analyses showed that male and female current smokers and men and women living in difficult conditions were likely to have a lower prudent dietary pattern score; male current smokers and male daily alcohol drinkers were likely to have a higher meat dietary pattern score.

**Conclusions:**

During the year after the earthquake, the prudent dietary pattern was associated with better living conditions among survivors, whereas the meat dietary pattern was not.

## Abstract (ja)

**背景：:**

2011年3月11日の東日本大震災からの1年間に、被災者の健康状態や生活習慣が広範囲に調査された。本研究は被災者の暮らし向きと食事パターンの関連を明らかにすることを目的とした。

**方法：:**

岩手県の調査地域において、同年齢の人口の25％に相当する18歳以上の10,466人が参加した。質問票により、8食品群について毎日の平均摂取頻度の回答を得た。対象者の85％が3回摂取していた主食を除き、7食品群について9,789人（男性3,795人、女性5,994人）を対象に因子分析を行った。

**結果：:**

因子分析により、健康志向と肉食志向の2つの食事パターンが同定された。魚・貝等、豆腐・納豆等、野菜、くだもの、牛乳・ヨーグルト・チーズ等の高摂取頻度で特徴付けられる健康志向パターンは、高齢の者及び女性において顕著であった。肉と卵の高摂取頻度で特徴付けられる肉食志向パターンは、若年の者及び男性において顕著であった。年齢調整した多変量ロジスティック回帰分析の結果、男女とも現在喫煙者と暮らし向きが苦しい者において健康志向パターンの得点が低い者が多く、男性では現在喫煙者と毎日飲酒者において肉食志向パターンの得点が高い者が多かった。

**結論：:**

大震災後1年間の被災者では、健康志向パターンは暮らし向きと関連していたが、肉食志向パターンではその関連はみられなかった。

## INTRODUCTION

Due to concerns regarding the physical and mental well-being of survivors of the Great East Japan Earthquake and tsunami disaster of March 11, 2011, a prospective cohort was established to address an urgent need to monitor their health. During the year after the disaster, health examinations and questionnaires were used to assess the health and living conditions of survivors as a baseline survey for a follow-up study. The survey in Iwate Prefecture was conducted among survivors living mainly in temporary housing in 2 cities and 2 towns on the Pacific coast.^1^

Regarding lifestyle, the quantity and quality of food intake seriously deteriorated among survivors during the first few months after the disaster, when most were living in evacuation centers.^2^ After about half a year, the survivors were transferred to temporary accommodation, where only basic necessities were supplied by local governments, and they began preparing their own food. However, because their economic circumstances had not changed and a number had lost family members who had played a major role in preparing food, it is likely that dietary conditions remained poor.

In developing a common questionnaire for a baseline survey of the survivors, we broadly investigated their health conditions and lifestyles. With respect to food intake, space limitations on the questionnaire necessitated the use of a short food frequency questionnaire (FFQ) instead of an established comprehensive FFQ. With regard to major nutrients in the standard Japanese diet, 8 food groups were selected, and the frequency of consumption of these food groups during the previous several days was assessed in the questionnaire.

The purpose of this study was to investigate the dietary patterns of survivors of the Great East Japan Earthquake and examine the relationship between these patterns and their living conditions.

## METHODS

### Participants

From September 2011 to February 2012, 10 466 people aged 18 years or older, (25% of the population of that age in the area) participated in the Health Survey of Great East Japan Earthquake survivors in Rikuzentakata city (from October 2011 to December 2011, and in February 2012), Kamaishi city (in October 2011), Yamada town (from September 2011 to November 2011), and Otsuchi town (in December 2011) on the Pacific coast of Iwate Prefecture. The survey was conducted by means of health checkups and a common questionnaire inquiring about health conditions and lifestyles.

### Health checkups and questionnaire

In developing the questionnaire, we searched the literature to identify a short questionnaire that addressed nutritional intake among Japanese. After omitting 2 food groups (confectionary and soft drinks) from the list of food groups on the questionnaire of Yatsuya et al^3^ and adding 2 groups (staple food and soybean products), we selected 8 food groups to investigate among the earthquake disaster survivors: staple food items (rice, bread, noodles), meat, fish and shellfish, eggs, soybean products (tofu and *natto* [fermented soybeans]), vegetables, fruit, and dairy products (milk, yogurt, cheese). Questions asked about the frequency of consumption (ie, the approximate number of times a day on average during the previous several days) for these 8 food groups. The response categories were none (<1 time), once, twice, 3 times, and 4 times or more. For simplicity, details on portion sizes were not investigated.

Living conditions were addressed by asking the question: “How do you feel about your current economic situation?”. Participants were requested to select an answer from 1 of 4 options: severe, difficult, rather difficult, and acceptable. Data relating to smoking and drinking habits were obtained from a lifestyle sheet, the information for which was obtained during the health checkup. The options for smoking were nonsmoker, former smoker, and current smoker. The options for alcohol consumption were none, occasional, and daily.

Physical examinations and blood testing were conducted during the health checkups. Height and weight were measured, and body mass index (BMI) was calculated as weight (kilograms) divided by the square of the height (meters). After a 5-minute rest, blood pressure was consecutively measured twice while the participant was seated. All measurements were performed by well-trained staff using automatic devices, and the average of the 2 measurements was used for statistical analysis.

### Statistical analysis

After excluding staple foods, which were consumed 3 times a day by 85% of the participants, factor analysis using the maximum likelihood method with varimax rotation was conducted on 7 food groups among 9789 individuals (3795 men and 5994 women). Factors with an eigenvalue greater than 1 were extracted. Food groups whose factor loadings were greater than 0.4 were used to define factors. Factor scores were calculated for all participants, and dichotomous variables were made by separating participants with values greater than or equal to the mean from those with values below the mean.

Multiple logistic regression analyses were performed by sex, with dichotomous variables of the factor score as dependent variables and age (≤44 years [reference], 45–54, 55–64, 65–74, ≥75 years), living conditions (acceptable [reference], rather difficult, difficult/severe), smoking habit (none [reference], past, current), and drinking habit (none [reference], occasionally, daily) as independent variables. All analyses were conducted using IBM SPSS Statistics version 19.0 (Tokyo, Japan), and *P* values less than 0.05 were regarded as statistically significant.

### Ethical considerations

This study was approved by the ethics committees of the National Institute of Health and Nutrition and Iwate Medical University.

## RESULTS

Table 1 shows the baseline characteristics of the participants. Around 30% of men and women were in the age group 65 to 74 years. The prevalence of current smoking was about 30% among men and less than 10% among women. The prevalence of daily alcohol consumption was about 40% among men and 5% among women. Almost 50% of both men and women answered that their living conditions were acceptable, but more than 20% answered that their living conditions were difficult or severe.

**Table 1.** Baseline characteristics of participants

	Men (*n* = 3795)	Women (*n* = 5994)
Age group (years)		
≤44	586 (15.4%)	1073 (17.9%)
45–54	390 (10.3%)	757 (12.6%)
55–64	886 (23.3%)	1579 (26.3%)
65–74	1224 (32.3%)	1690 (28.2%)
≥75	709 (18.7%)	895 (14.9%)
Smoking habit		
None	1386 (36.5%)	5388 (89.9%)
Past	1235 (32.5%)	175 (2.9%)
Current	1174 (30.9%)	429 (7.2%)
Unknown	0 (0%)	2 (0.0%)
Drinking habit		
None	1471 (38.8%)	5097 (85.0%)
Occasionally	782 (20.6%)	628 (10.5%)
Daily	1542 (40.6%)	267 (4.5%)
Unknown	0 (0%)	2 (0.0%)
Living conditions		
Acceptable	1781 (46.9%)	2961 (49.4%)
Rather difficult	1033 (27.2%)	1706 (28.5%)
Difficult	670 (17.7%)	910 (15.2%)
Severe	295 (7.8%)	401 (6.7%)
Unknown	16 (0.4%)	16 (0.3%)

For the 8 food groups, the percentages for the average number of times of daily consumption are shown in Table 2. The highest percentage was once daily in all food groups—except for staple food items and vegetables, which were most often consumed 3 times a day. Because 85% of participants consumed staple foods 3 times a day, which skewed the distribution, this food group was excluded from factor analysis.

**Table 2.** Average daily frequency of food group consumption (*n* = 9789)

Food group	Number of times per day				

	0	1	2	3	≥4
Staple food items (rice, bread, noodles) (*n* = 9778)	0.1%	2.9%	10.6%	85.4%	1.0%
Meat	25.1%	63.4%	9.8%	1.5%	0.2%
Fish and shellfish	5.7%	62.9%	24.7%	5.8%	0.8%
Eggs	14.6%	72.2%	10.2%	2.5%	0.5%
Soybean products (tofu, natto)	5.0%	55.2%	27.1%	11.5%	1.2%
Vegetables	0.9%	20.3%	34.3%	41.8%	2.8%
Fruit	10.9%	50.1%	25.7%	11.6%	1.7%
Milk products (milk, yogurt, cheese)	17.3%	57.9%	16.7%	6.6%	1.5%

Using the remaining 7 food groups, we conducted factor analysis with the maximum likelihood method and varimax rotation; the factor loadings are shown in Table 3. Two factors with eigenvalues greater than 1 were extracted. The first factor was characterized by fish and shellfish, soybean products, vegetables, fruit, and dairy products; the second factor was characterized by meat and eggs. We termed the first factor the prudent dietary pattern and the second the meat dietary pattern.

**Table 3.** Factor loadings for prudent and meat dietary patterns (*n* = 9789)

Food group	Dietary pattern	

	Prudent	Meat
Meat	0.07	0.54
Fish and shellfish	0.41	0.35
Eggs	0.24	0.66
Soybean products (tofu, natto)	0.55	0.29
Vegetables	0.63	0.08
Fruit	0.62	0.09
Milk products (milk, yogurt, cheese)	0.43	0.24

The proportions of participants with a factor score greater than or equal to the mean for the prudent and meat dietary patterns, by sex and age group, are shown in Table 4. The prudent dietary pattern was more prevalent among women than among men in each age group; it was also more prevalent among older participants. In contrast, the meat dietary pattern was more prevalent among men than among women, in each age group, and prevalence was lower among older participants.

**Table 4.** Prevalence of participants who had a factor score greater than or equal to the mean for the prudent and meat dietary patterns, by sex and age group (*n* = 9789)

Age group (years)	Prudent dietary pattern		Meat dietary pattern	
	
	Men (*n* = 3795)	Women (*n* = 5994)	Men (*n* = 3795)	Women (*n* = 5994)
≤44	21.5%	29.2%	73.9%	58.2%
45–54	26.4%	37.3%	62.8%	50.7%
55–64	38.0%	55.4%	53.4%	40.1%
65–74	49.2%	65.0%	43.5%	35.7%
≥75	54.3%	63.6%	39.6%	33.4%

Using multiple logistic regression analyses, odds ratios and 95% CIs of the factor scores greater than or equal to the mean for the prudent and meat dietary patterns were calculated for men and women (Table 5). Age was positively associated with a higher score for the prudent dietary pattern among men and women and inversely associated with a higher score for the meat dietary pattern among men and women. Male current smokers and female former and current smokers were not likely to have a higher score for the prudent dietary pattern; male current smokers were likely to have a higher score for the meat dietary pattern. Male daily alcohol drinkers were not likely to have a higher score for the prudent dietary pattern; male daily drinkers and female occasional drinkers were likely to have a higher score for the meat dietary pattern. Both men and women whose living conditions were rather difficult, difficult, or severe were not likely to have a higher score for the prudent dietary pattern, but living conditions were not associated with the meat dietary pattern. The association between living conditions and frequency of intake of each food group (excluding staple foods) was also examined. When adjusted for age, regression analyses showed statistically significant associations for the following: more-difficult living conditions were associated with less frequent intake of vegetables (*P* < 0.001), fruit (*P* < 0.001), and milk products (*P* < 0.001) among men, and less frequent intake of meat (*P* = 0.010), fish and shellfish (*P* = 0.025), vegetables (*P* < 0.001), fruit (*P* < 0.001), and milk products (*P* < 0.001) among women (data not shown).

**Table 5.** Odds ratios (OR) and 95% CIs for factor scores greater than or equal to the mean for the prudent and meat dietary patterns on multiple logistic regression analyses (*n* = 9754)^a^

	Prudent dietary pattern		Meat dietary pattern	
		
	Men (*n* = 3779)	Women (*n* = 5975)	Men (*n* = 3779)	Women (*n* = 5975)
				
	OR (95% CI)	OR (95% CI)	OR (95% CI)	OR (95% CI)
Age (years)^b^				
18–44	1.00 (reference)	1.00 (reference)	1.00 (reference)	1.00 (reference)
45–54	1.35 (1.05–1.65)	1.34 (1.14–1.54)	0.58 (0.30–0.86)	0.74 (0.55–0.92)
55–64	2.25 (2.01–2.49)	2.64 (2.47–2.81)	0.40 (0.17–0.63)	0.48 (0.32–0.65)
65–74	3.35 (3.11–3.58)	3.79 (3.62–3.97)	0.29 (0.06–0.51)	0.41 (0.24–0.58)
≥75	3.84 (3.58–4.09)	3.46 (3.26–3.66)	0.26 (0.01–0.51)	0.37 (0.18–0.57)
Smoking habit				
None	1.00 (reference)	1.00 (reference)	1.00 (reference)	1.00 (reference)
Past	0.93 (0.77–1.09)	0.51 (0.16–0.86)	0.97 (0.81–1.13)	0.94 (0.62–1.25)
Current	0.81 (0.64–0.98)	0.54 (0.30–0.78)	1.25 (1.08–1.42)	0.95 (0.73–1.16)
Drinking habit				
None	1.00 (reference)	1.00 (reference)	1.00 (reference)	1.00 (reference)
Occasionally	1.00 (0.82–1.18)	0.94 (0.76–1.12)	1.07 (0.89–1.25)	1.21 (1.03–1.38)
Daily	0.84 (0.68–0.99)	0.88 (0.60–1.16)	1.25 (1.10–1.41)	1.20 (0.93–1.46)
Living conditions				
Acceptable	1.00 (reference)	1.00 (reference)	1.00 (reference)	1.00 (reference)
Rather difficult	0.78 (0.62–0.95)	0.87 (0.75–1.00)	0.93 (0.77–1.09)	1.11 (0.98–1.23)
Difficult/severe	0.80 (0.63–0.97)	0.83 (0.69–0.97)	1.13 (0.97–1.30)	0.97 (0.83–1.10)

^a^Fewer participants, due to missing observations. ^b^All variables in model are listed in the table.

## DISCUSSION

In a baseline survey of the cohort study of survivors of the Great East Japan Earthquake, prudent and meat dietary patterns were extracted from a short list of food groups in the questionnaire. Women and older participants tended to adopt the prudent dietary pattern, whereas men and younger participants tended to follow the meat dietary pattern. After adjustment for age, living conditions were associated with the prudent dietary pattern, and with intakes of vegetables, fruit, and milk, when analyzed by food group, among men and women. These findings may reflect the fact that economic conditions were a key factor in the ability of survivors to maintain a healthy diet 1 year after the disaster.

Living conditions are considered a sociodemographic indicator. Several studies have found a relationship between sociodemographic factors and dietary patterns.^4^^–^^6^ The Multiethnic Cohort Study, conducted in Hawaii and Los Angeles from 1993 to 1996, investigated participants from 5 ethnic groups (African-Americans, Hawaiians, Japanese-Americans, Latinos, and whites). The authors identified “fat and meat,” “vegetables,” and “fruit and milk” dietary patterns and found a weak inverse association between education and the fat and meat dietary pattern, and weak positive associations of education with the vegetables and fruit and milk dietary patterns.^5^ In a middle-aged Japanese population, Sadakane et al identified vegetable, meat, and Western dietary patterns: the vegetable pattern corresponded to the prudent dietary pattern in the present study, and the Western dietary pattern was the reverse of the present traditional Japanese dietary pattern.^6^ The authors found that the meat pattern and Western pattern (among men) and the vegetable pattern, meat pattern, and Western pattern (among women) were positively associated with duration of education. These results partly differed from those of the present study, but it should be noted that there was no adjustment for age in the study by Sadakane et al.

With regard to smoking, an inverse relationship between the prudent dietary pattern and current smoking was observed among both men and women in the present study; however, the relationship between the meat dietary pattern and current smoking differed between men and women. For alcohol consumption, we noted inverse and positive relationships of daily drinking with the prudent and meat dietary patterns, respectively. However, these relationships were statistically significant only among men, perhaps because the percentage of daily alcohol consumption was low among women (4.5%). In a community-based case-control study of colorectal cancer risk in Japan, Kurotani et al found an inverse association of ever-smoking with the prudent pattern but not the high-fat pattern, which resembled the meat dietary pattern in the present study.^7^ The absence of an association of alcohol consumption with the prudent or high-fat patterns may have been because such consumption was more common (>75% among men and >25% among women) in their study.

In the Multiethnic Cohort Study, dietary pattern was significantly associated with BMI.^5^ In our study, BMI was not associated with dietary pattern among men or women. In addition, in the present study, systolic blood pressure, HbA1c, and total cholesterol (markers of hypertension, diabetes, and hyperlipidemia, respectively) were not related to dietary pattern among men or women. Because living conditions are closely related to subjective health, we repeated our analyses by adding subjective health to the multiple logistic regression models; however, the results did not materially differ from those of the main analysis.

We used 7 food groups in the factor analysis, and only 2 dietary patterns were extracted. Previous studies of the Japanese diet have generally identified 3 dietary patterns: the prudent, Westernized, and traditional Japanese dietary patterns.^7^^–^^9^ The prudent dietary pattern is characterized by high intakes of vegetables, fruits, seafood, and soy foods; the Westernized pattern is characterized by high intakes of fat and oils, red meat, processed meat, and mayonnaise; the traditional Japanese dietary pattern is characterized by high intakes of rice, miso soup, and pickles. The 2 dietary patterns identified in our study correspond to 2 of the above 3 dietary patterns—prudent and Westernized. Our failure to identify the traditional Japanese dietary pattern was probably due to the fact that staple foods were excluded from factor analysis, and because miso soup and pickles were not listed on the questionnaire.

This study had limitations. First, the short list of food groups on the questionnaire could not capture intakes of some food groups, such as miso soup and pickles, which could have formed part of another dietary pattern—the traditional Japanese dietary pattern. Intakes of these food groups need to be carefully monitored because of the possibility of higher salt intake when these food groups are excessively consumed. However, the main aim of this survey was to detect insufficient intake of the major food groups. Thus, instead of adopting a comprehensive food frequency questionnaire, a short list of food groups was used on the questionnaire. Second, causal relationships cannot be inferred from the results of this study because of its cross-sectional nature. Third, with regard to the second point, dietary patterns were identified a posteriori, and the associations observed among the study participants only pertain to the year after the earthquake. Although there may have been an association between dietary patterns and living conditions before the earthquake, it is possible that the disaster accentuated this relationship. The participants are currently being monitored in the cohort study,^1^ and the present associations will be investigated over a longer period.

In conclusion, during the year after the Great East Japan Earthquake on March 11, 2011, the health conditions and lifestyles of survivors were widely surveyed. The prudent dietary pattern—1 of 2 dietary patterns identified in the short list of food groups—was associated with better living conditions. Future studies should examine food availability in local stores and transport methods and identify the key factors that help those living in difficult conditions to adopt better dietary habits. Prospective observations of the cohort must also attempt to determine if the prudent dietary pattern is associated with better health outcomes.

## ONLINE ONLY MATERIALS

Abstract in Japanese.
