# Supplementary material for: Relationship of Living Conditions With Dietary Patterns Among Survivors of the Great East Japan Earthquake
Source: J Epidemiol. 2013 Sep 5;23(5):376–81. doi: 10.2188/jea.JE20130025 (PMC3775532; doi:10.2188/jea.JE20130025)
Supplement: Abstract in Japanese. [file je-23-376-s001.pdf]

東日本大震災被災者における暮らし向きと食事パターンの関連

西　信雄,[1] 吉村英一,[2] 髙田和子,[2] 笠岡（坪山）宜代,[3] 窪田哲也,[4] 宮地元彦,[5]
徳留信寛,[6] 横山由香里,[7] 坂田清美,[7] 小林誠一郎,[8] 小川　彰[9]

[1]（独）国立健康・栄養研究所　国際産学連携センター
[2]（独）国立健康・栄養研究所　栄養教育研究部
[3]（独）国立健康・栄養研究所　栄養疫学研究部
[4]（独）国立健康・栄養研究所　臨床栄養研究部
[5]（独）国立健康・栄養研究所　健康増進研究部
[6]（独）国立健康・栄養研究所
[7]岩手医科大学医学部衛生学公衆衛生学
[8]岩手医科大学医学部形成外科学
[9]岩手医科大学

抄録
背景 : 2011 年 3 月 11 日の東日本大震災からの 1 年間に、被災者の健康状態や生活習慣が広範囲に調査された。本研究は被災者の暮らし向きと食事パターンの関連を明らかにすることを目的とした。
方法 : 岩手県の調査地域において、同年齢の人口の 25%に相当する 18 歳以上の 10,466 人が参加した。質問票により、8 食品群について毎日の平均摂取頻度の回答を得た。対象者の 85%が 3 回摂取していた主食を除き、7 食品群について 9,789 人（男性 3,795 人、女性 5,994 人）を対象に因子分析を行った。
結果 : 因子分析により、健康志向と肉食志向の 2 つの食事パターンが同定された。魚・貝等、豆腐・納豆等、野菜、くだもの、牛乳・ヨーグルト・チーズ等の高摂取頻度で特徴付けられる健康志向パターンは、高齢の者及び女性において顕著であった。肉と卵の高摂取頻度で特徴付けられる肉食志向パターンは、若年の者及び男性において顕著であった。年齢調整した多変量ロジスティック回帰分析の結果、男女とも現在喫煙者と暮らし向きが苦しい者において健康志向パターンの得点が低い者が多く、男性では現在喫煙者と毎日飲酒者において肉食志向パターンの得点が高い者が多かった。
結論 : 大震災後 1 年間の被災者では、健康志向パターンは暮らし向きと関連していたが、肉食志向パターンではその関連はみられなかった。

キーワード : 東日本大震災、暮らし向き、食事パターン
